# Assessing real-world safety of plecanatide: a pharmacovigilance study based on the FDA adverse event reporting system

**DOI:** 10.3389/fphar.2024.1500810

**Published:** 2024-11-25

**Authors:** Zhiyuan Zhang, Yifan Yao, Li Zhu

**Affiliations:** ^1^ The Second Clinical College of Dalian Medical University, Dalian Medical University, Dalian, Liaoning, China; ^2^ The Affiliated Taizhou People’s Hospital of Nanjing Medical University, Taizhou School of Clinical Medicine, Nanjing Medical University, Taizhou, Jiangsu, China

**Keywords:** plecanatide, disproportionality analysis, CIC, IBS-C, FAERS

## Abstract

**Background:**

Plecanatide is a selective gastrointestinal peptide used for the treatment of chronic idiopathic constipation (CIC) and irritable bowel syndrome with constipation (IBS-C). Given its widespread use, understanding the long-term safety of plecanatide in real-world settings is essential.

**Methods:**

Data for this study were sourced from the United States Food and Drug Administration (FDA) Adverse Event Reporting System (FAERS) from the first quarter of 2017 to the second quarter of 2024. Disproportionality analysis methods were employed to quantify adverse event signals associated with plecanatide. Additionally, a Weibull distribution analysis was conducted to assess changes in adverse events over time. Sensitivity analyses were performed to enhance the robustness of the findings.

**Results:**

A total of 861 cases associated with plecanatide were identified, including 2057 adverse event reports. Common positive adverse events included diarrhea, constipation, abdominal distension, dissatisfaction with treatment, rectal tenesmus, increased fecal volume, abnormal gastrointestinal sounds, and gastrointestinal motility disorders. The majority of adverse events related to plecanatide occurred within the first 7 days of treatment. Findings were consistent across sensitivity analyses.

**Conclusion:**

This study preliminarily explores the safety of plecanatide in real-world applications, revealing significant new adverse event signals. These findings provide important safety references for clinicians prescribing plecanatide for CIC and IBS-C.

## 1 Introduction

Plecanatide is a 16-amino acid peptide that serves as an agonist of guanylate cyclase-C (GC-C). Plecanatide binds to GC-C and locates on the apical surface of intestinal epithelial cells, thereby leading to an increase in both intracellular and extracellular cyclic guanosine monophosphate (cGMP) levels. The elevated cGMP activates the cystic fibrosis transmembrane conductance regulator (CFTR) ion channel, promoting chloride and bicarbonate secretion into the intestinal lumen, which increases intestinal fluid and accelerates transit (Al-Salama and Syed, 2017).

Plecanatide was first developed in 2007 and received the approval from the United States Food and Drug Administration (FDA) in January 2017 for the treatment of adult chronic idiopathic constipation (CIC). In 2018, the drug was further approved for the indication of irritable bowel syndrome with constipation (IBS-C) (Brenner et al., 2018). While typically not life-threatening, both CIC and IBS-C are common chronic diseases that contribute to significant healthcare costs, reduced quality of life, and the occurrence of psychological comorbidities (Chang et al., 2022; Chang et al., 2023). According to previous studies, the updated guidelines recommend the prescription of plecanatide for adult individuals with CIC and recommend plecanatide for the treatment of IBS-C (Chang et al., 2022; Chang et al., 2023).

Various clinical trials have confirmed the efficacy and safety of plecanatide for the treatment of CIC and IBS-C (Miner et al., 2017; Brenner et al., 2018). Following treatment with plecanatide, patients with CIC experienced improvements in both complete spontaneous bowel movement (CSBM) and spontaneous bowel movements (SBM) frequencies, while a significantly higher proportion of IBS-C patients experienced at least a 30% improvement in the severity of their most severe abdominal pain. The most frequently reported adverse event associated with plecanatide was diarrhea, with an incidence of approximately 4% (Brenner et al., 2023; Rao et al., 2023).

The incidence of CIC and IBS-C is relatively high in the United States, with estimated prevalence rates of 12% for CIC and 10%–14% for IBS-C (Suares and Ford, 2011; Ford et al., 2018). As plecanatide is indicated for the treatment of these conditions, its use is on the rise (Love, 2019). Additionally, plecanatide has been prescribed across a broad range of individuals, including children (Wolfson and Saps, 2024), older adults (Menees et al., 2020), and those with chronic kidney disease (Cha et al., 2023). Given the limited real-world safety data on plecanatide, a pharmacovigilance study is essential.

Established by the FDA since the 1960s, the FDA Adverse Event Reporting System (FAERS) plays a key role in post-marketing surveillance of adverse events. FAERS receives reports of potential adverse drug reactions from consumers, pharmacists, and clinicians (Gendreau and Potenza, 2014). The large data volume, combined with public accessibility, makes FAERS a widely utilized tool for evaluating the safety of various drugs in real-world settings, facilitating transparent and comprehensive safety assessments (Chen et al., 2024; Dou et al., 2024).

This study aims to evaluate the real-world safety of plecanatide using data from the FAERS database.

## 2 Methods

### 2.1 Study design and data source

The FAERS is a database designed to support the FDA’s post-marketing surveillance program for drugs and therapeutic biological products. Data in the FAERS database is voluntarily submitted by physicians, pharmacists, consumers, manufacturers, and other sources (Hua et al., 2024). We conducted a retrospective pharmacovigilance study on adverse events related to plecanatide, utilizing data from the FAERS database (https://open.fda.gov/data/faers/) from the first quarter of 2017 (plecanatide was approved in January 2017) to the second quarter of 2024. The FAERS database consists of seven components: demographics (DEMO), drug information (DRUG), adverse events (REAC), patient outcomes (OUTC), report sources (RPSR), treatment duration (THE), and indications (INDI).

**FIGURE 1:**
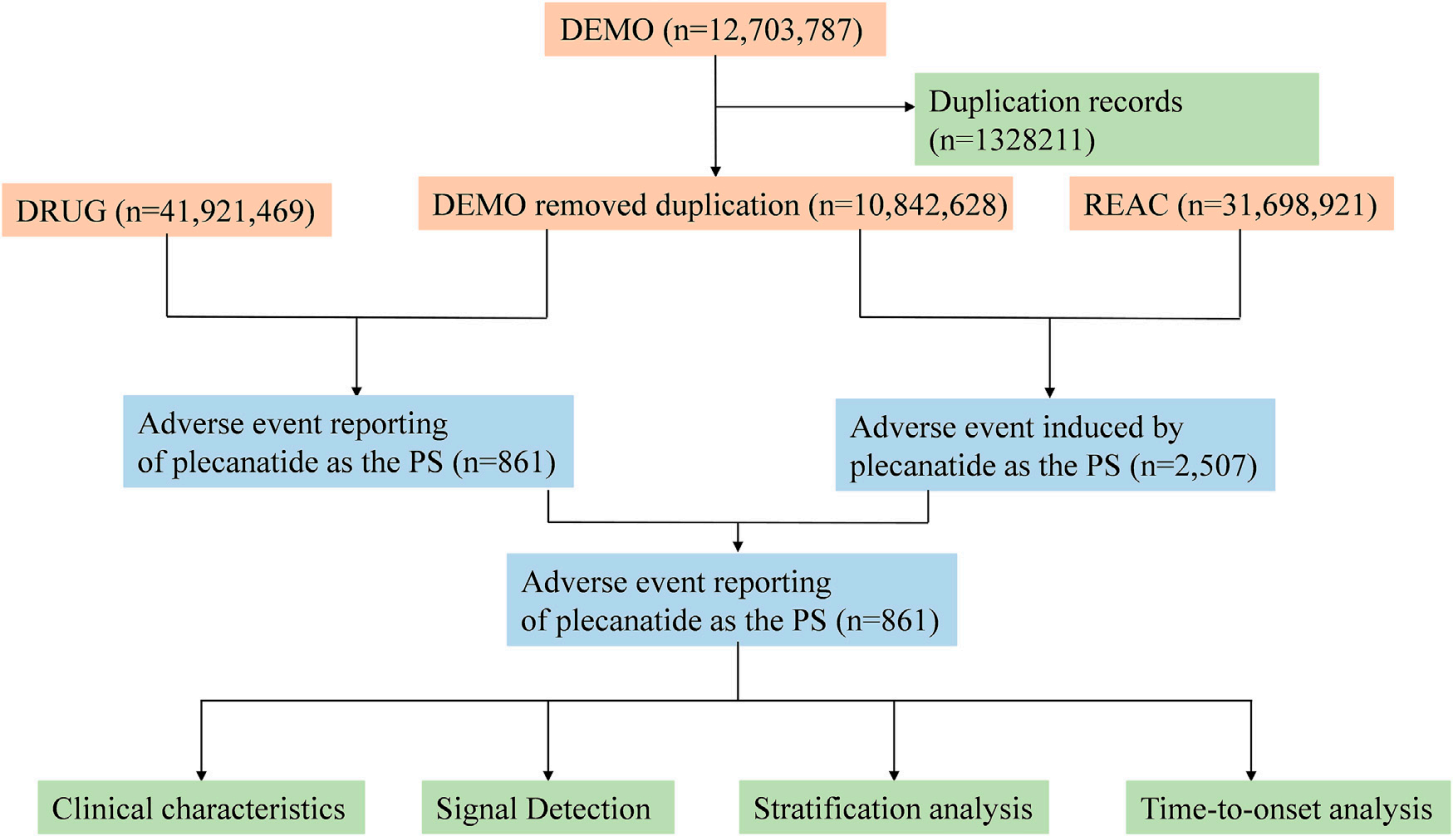
Flowchart of the adverse event analysis process for plecanatide using the FAERS database.

To identify the target drug in the FAERS database, a comprehensive approach was employed. Specifically, this study utilized plecanatide (generic name), Trulance (brand name), and plecanatide acetate (active ingredient) as primary suspect (PS) terms to examine plecanatide-related adverse events recorded in the FAERS database. Following FDA recommendations, we performed data cleaning to remove duplicate reports. For cases with the same participant identifiers (CASEIDs), the report with the highest FDA report receipt date (FDA_DT) was selected. For cases with identical CASEID and FDA_DT, the report with the highest unique report identifier (PRIMARYID) was retained. Adverse event terms were standardized using the preferred terms (PTs) from MedDRA. Both PT and system organ class (SOC) were sourced from Medical Dictionary for Regulatory Activities (MedDRA) (Version 26.1) for further analysis. The detailed study process is depicted in Figure 1.

### 2.2 Statistical analysis

#### 2.2.1 Disproportionality analysis

This pharmacovigilance study employed descriptive and disproportionality analyses to investigate whether specific adverse events were associated with plecanatide. Disproportionality analysis is widely regarded as a crucial method for identifying drug-related safety signals. Four key algorithms were used to detect potential signals related to plecanatide: reporting odds ratio (ROR), proportional reporting ratio (PRR), multi-item gamma Poisson shrinker (MGPS), and Bayesian confidence propagation neural network (BCPNN) (Jin et al., 2024; Wu et al., 2024). All four methods, detailed in Supplementary Table S1, relied on two-by-two contingency tables. The detailed algorithms and thresholds of four methods were exhibited in Supplementary Table S2. Adverse events that met all of the four method thresholds were considered potential signals. All analyses were performed using R software (version 4.4.1).

#### 2.2.2 Weibull distribution analysis

The time to onset of plecanatide-related adverse events was defined as the interval between the start of plecanatide use and the occurrence of the adverse event. To assess changes in adverse events over time, a Weibull distribution analysis was performed. The shape parameter (β) was used to assess the failure rate over time. When β < 1 and the 95% confidence interval (CI) is also <1, this indicates an early failure model, where the adverse event rate is higher shortly after treatment initiation and decreases over time. When β = 1 and the 95% CI includes 1, it suggests a constant failure rate, indicating that the risk of adverse events remains stable throughout the treatment period. Conversely, when β > 1 and the 95% CI does not include 1, this represents a wear-out failure model, where the adverse event rate increases over time, suggesting a statistically significant rise in risk with prolonged treatment.

#### 2.2.3 Sensitivity analysis

To more accurately assess the independent safety profile of plecanatide in real-world settings, a stepwise sensitivity analysis was conducted, excluding adverse event reports involving the top three and top five most commonly co-administered drugs. The progressive exclusion of drugs frequently co-administered with plecanatide helped reduce confounding from similar or overlapping adverse events and control for potential cross-system interactions. This stepwise method effectively isolated the independent effects of plecanatide, thereby improving the accuracy of signal detection and the reliability of the study findings.

## 3 Results

### 3.1 Descriptive characteristics

A total of 861 individual case safety reports (ICSRs) associated with plecanatide were detected, encompassing 2057 adverse events spanning from 2017 Q1 to 2024 Q2. Clinical characteristics of these reports were displayed in Table 1. Of the cases, female and male accounted for 48.3% and 16.4%, respectively. Patients aged ≥45 years comprised the predominant age group, accounting for 22.6%. The majority of reports were non-healthcare professionals, consisting of 69.5%. The most common indication of plecanatide was constipation and irritable bowel syndrome. Miralax, omeprazole, linzess, Synthroid, and lisinopril were the top 5 co-administered medications with plecanatide.

**TABLE 1:** Clinical characteristics of reports associated with plecanatide (2017 Q1-2024 Q2).

Characteristics	Number of cases	Proportion of cases (%)
Number of adverse events	861	
Year of report		
2017	140	16.3
2018	162	18.1
2019	135	15.7
2020	123	14.3
2021	121	14.1
2022	63	7.3
2023	77	8.9
2024	40	4.6
Gender		
Female	416	48.3
Male	141	16.4
Unknown	304	35.3
Age		
<18	1	0.1
18–44	47	5.5
44–64	100	11.6
≥65	195	22.6
Unknown	518	60.2
Weight, kg		
<80	111	12.9
80–100	28	3.3
>100	16	1.9
Unknown	706	82.0
Reporter		
Non-healthcare professional	598	69.5
Healthcare professional	260	30.2
Unknown	3	0.3
Reported countries		
United States	855	99.3
Canada	1	0.1
Other	5	0.6
Serious outcome		
Death	35	4.1
Life threatening	4	0.5
Hospitalization	37	4.3
Disability	1	0.1
Other	68	7.9
Indication (Top 5)		
Constipation	448	52.0
Irritable bowel syndrome	170	19.7
Diverticulitis	4	0.5
Impaired gastric emptying	4	0.5
Gastrointestinal motility disorder	3	0.3
Medication combinations (Top 5)		
Miralax	64	7.4
Omeprazole	30	3.5
Linzess	30	3.5
Synthroid	29	3.4
Lisinopril	23	2.7

### 3.2 Adverse events at the SOC levels

**FIGURE 2:**
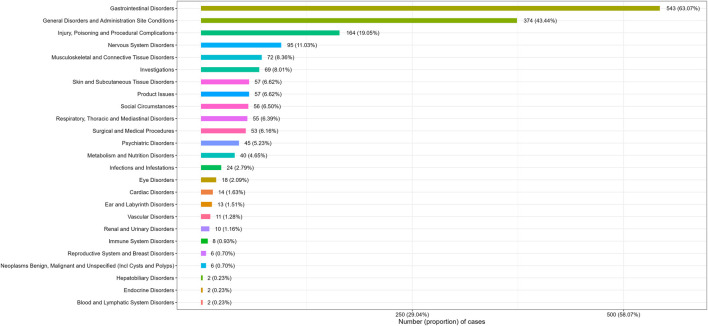
Number (proportion) of cases categorized by system organ class for plecanatide.

Adverse events at the SOC levels were demonstrated in Table 2. A total of 25 SOCs were associated with plecanatide. Positive signals were observed in gastrointestinal disorders, social circumstances, product issues, and surgical and medical procedures. As shown in Figure 2, the most common SOC with the highest signal strength was gastrointestinal disorders, with 543 (63.07%) cases identified.

**TABLE 2:** The signal strength of adverse event reporting related to plecanatide at the SOC level in FAERS database.

SOC	Case number	ROR (95% CI)	PRR (χ^2^)	IC (IC025)	EBGM (EBGM05)
Gastrointestinal disorders	543	5.73 (5.18–6.34)	4.3 (1,478.25)	4.3 (3.95)	2.1 (1.96)
General disorders and administration site conditions	374	1.22 (1.09–1.37)	1.18 (12.21)	1.18 (1.07)	0.24 (0.07)
Injury, poisoning and procedural complications	164	0.74 (0.63–0.87)	0.77 (13.4)	0.77 (0.67)	−0.39 (−0.62)
Nervous system disorders	95	0.7 (0.57–0.85)	0.71 (12.02)	0.71 (0.6)	−0.49 (−0.79)
Musculoskeletal and connective tissue disorders	72	0.9 (0.71–1.14)	0.91 (0.71)	0.91 (0.74)	−0.14 (−0.48)
Investigations	69	0.69 (0.54–0.88)	0.7 (9.13)	0.7 (0.58)	−0.51 (−0.86)
Skin and subcutaneous tissue disorders	57	0.58 (0.45–0.76)	0.59 (16.65)	0.59 (0.48)	−0.75 (−1.14)
Product issues	57	1.5 (1.15–1.95)	1.49 (9.23)	1.48 (1.19)	0.57 (0.19)
Social circumstances	56	5.51 (4.22–7.19)	5.37 (200.31)	5.37 (4.3)	2.42 (2.04)
Respiratory, thoracic and mediastinal disorders	55	0.7 (0.54–0.92)	0.71 (6.86)	0.71 (0.57)	−0.5 (−0.89)
Surgical and medical procedures	53	1.65 (1.25–2.17)	1.63 (13.12)	1.63 (1.3)	0.7 (0.31)
Psychiatric disorders	45	0.51 (0.38–0.69)	0.53 (20.18)	0.53 (0.41)	−0.93 (−1.36)
Metabolism and nutrition disorders	40	0.95 (0.69–1.3)	0.95 (0.11)	0.95 (0.73)	−0.07 (−0.53)
Infections and infestations	24	0.23 (0.15–0.34)	0.24 (61.98)	0.24 (0.17)	−2.07 (−2.65)
Eye disorders	18	0.54 (0.34–0.85)	0.54 (7.16)	0.54 (0.37)	−0.89 (−1.55)
Cardiac disorders	14	0.34 (0.2–0.57)	0.34 (17.99)	0.34 (0.22)	−1.54 (−2.29)
Ear and labyrinth disorders	13	1.35 (0.78–2.33)	1.35 (1.16)	1.35 (0.85)	0.43 (−0.35)
Vascular disorders	11	0.25 (0.14–0.45)	0.26 (24.36)	0.26 (0.16)	−1.97 (−2.8)
Renal and urinary disorders	10	0.27 (0.14–0.49)	0.27 (20.23)	0.27 (0.16)	−1.89 (−2.76)
Immune system disorders	8	0.27 (0.14–0.54)	0.28 (15.51)	0.28 (0.15)	−1.86 (−2.83)
Reproductive system and breast disorders	6	0.44 (0.2–0.97)	0.44 (4.35)	0.44 (0.22)	−1.19 (−2.28)
Neoplasms benign, malignant and unspecified (incl cysts and polyps)	6	0.09 (0.04–0.19)	0.09 (58.85)	0.09 (0.05)	−3.5 (−4.6)
Hepatobiliary disorders	2	0.11 (0.03–0.46)	0.12 (13.68)	0.12 (0.04)	−3.11 (−4.78)
Blood and lymphatic system disorders	2	0.06 (0.01–0.23)	0.06 (30.22)	0.06 (0.02)	−4.07 (−5.74)
Endocrine disorders	2	0.34 (0.08–1.35)	0.34 (2.6)	0.34 (0.11)	−1.56 (−3.23)

SOC, system of organ; ROR, reporting odds ratio; PRR, proportional reporting ratio; EBGM, empirical Bayesian geometric mean; EBGM05, the lower limit of the 95% CI, of EBGM; IC, information component; IC025, the lower limit of the 95% CI, of the IC; CI, confidence interval.

### 3.3 Adverse events at the PT levels

Adverse events related with plecanatide at PT levels were sorted by ROR values and assessed for positive signals. Table 3 exhibited the top 50 plecanatide-related adverse events with positive signals ranked by ROR values at the preferred term (PT) level. Common adverse events at PT levels incorporated diarrhoea, constipation, abdominal distension, patient dissatisfaction with treatment, rectal tenesmus, faecal volume increased, gastrointestinal sounds abnormal, gastrointestinal motility disorder. Additionally, all adverse events with positive signals were shown in Supplementary Table S3.

**TABLE 3:** Top 50 plecanatide-related adverse events with positive signals rank by ROR values at the preferred term (PT) level.

PT	Case reports	ROR (95% Cl)	PRR (χ^2^)	EBGM (EBGM05)	IC (IC025)
Patient dissatisfaction with treatment	23	298.25 (196.89–451.8)	295.52 (6,597.08)	288.79 (204.02)	8.17 (6.5)
Rectal tenesmus	9	194.54 (100.6–376.21)	193.85 (1700.63)	190.94 (109.96)	7.58 (5.91)
Faecal volume increased	4	185.54 (69.09–498.31)	185.25 (722.43)	182.59 (79.89)	7.51 (5.84)
Gastrointestinal sounds abnormal	15	79.62 (47.85–132.48)	79.15 (1,150.35)	78.67 (51.38)	6.3 (4.63)
Gastrointestinal motility disorder	13	58.11 (33.65–100.34)	57.81 (722.55)	57.55 (36.44)	5.85 (4.18)
Dyschezia	9	52.31 (27.15–100.79)	52.12 (449.47)	51.91 (29.99)	5.7 (4.03)
Bowel movement irregularity	16	45.98 (28.1–75.24)	45.69 (697.04)	45.53 (30.16)	5.51 (3.84)
Faeces hard	7	42.48 (20.21–89.31)	42.37 (281.8)	42.23 (22.68)	5.4 (3.73)
Inability to afford medication	20	38.5 (24.78–59.82)	38.2 (722.54)	38.09 (26.34)	5.25 (3.58)
Defaecation urgency	11	37.82 (20.9–68.44)	37.66 (391.42)	37.55 (22.86)	5.23 (3.56)
Product packaging difficult to open	5	37.77 (15.69–90.94)	37.7 (178.09)	37.59 (18.02)	5.23 (3.56)
Abdominal tenderness	6	36.43 (16.33–81.27)	36.35 (205.68)	36.25 (18.52)	5.18 (3.51)
Flatulence	53	26.08 (19.86–34.24)	25.55 (1,248.51)	25.5 (20.3)	4.67 (3.01)
Haemorrhoidal haemorrhage	5	25.42 (10.56–61.17)	25.37 (116.81)	25.32 (12.14)	4.66 (2.99)
Anorectal discomfort	3	22.94 (7.39–71.26)	22.92 (62.77)	22.88 (8.86)	4.52 (2.85)
Gastrointestinal pain	10	22.15 (11.89–41.23)	22.06 (200.75)	22.02 (13.09)	4.46 (2.79)
Proctalgia	5	21.34 (8.87–51.35)	21.30 (96.58)	21.26 (10.2)	4.41 (2.74)
Symptom recurrence	7	21.26 (10.12–44.67)	21.20 (134.54)	21.17 (11.37)	4.40 (2.74)
Faeces discoloured	16	21.12 (12.91–34.53)	20.99 (304.14)	20.95 (13.88)	4.39 (2.72)
Abdominal distension	77	20.5 (16.33–25.72)	19.90 (1,381.91)	19.87 (16.43)	4.31 (2.65)
Anal incontinence	10	20.31 (10.91–37.81)	20.23 (182.53)	20.2 (12.01)	4.34 (2.67)
Faecaloma	4	19.74 (7.4–52.67)	19.71 (70.93)	19.68 (8.66)	4.30 (2.63)
Sinus headache	3	19.35 (6.23–60.1)	19.33 (52.07)	19.30 (7.48)	4.27 (2.6)
Functional gastrointestinal disorder	3	19.34 (6.23–60.07)	19.32 (52.05)	19.29 (7.48)	4.27 (2.6)
Insurance issue	12	17.4 (9.86–30.69)	17.32 (184.35)	17.3 (10.76)	4.11 (2.45)
Product solubility abnormal	4	16.11 (6.04–43)	16.09 (56.54)	16.07 (7.07)	4.01 (2.34)
Frequent bowel movements	17	14.43 (8.95–23.25)	14.34 (210.78)	14.32 (9.61)	3.84 (2.17)
Abnormal faeces	5	14.4 (5.99–34.65)	14.38 (62.17)	14.36 (6.89)	3.84 (2.18)
Therapeutic product effect variable	3	12.88 (4.15–40)	12.87 (32.81)	12.86 (4.98)	3.68 (2.02)
Diarrhoea	275	11.16 (9.84–12.64)	10.04 (2,261.73)	10.03 (9.04)	3.33 (1.66)
Polyp	3	10.91 (3.51–33.86)	10.9 (26.94)	10.89 (4.22)	3.44 (1.78)
Therapeutic product effect decreased	30	10.71 (7.47–15.35)	10.59 (260.59)	10.58 (7.83)	3.4 (1.74)
Thirst	7	10.19 (4.85–21.4)	10.16 (57.81)	10.16 (5.46)	3.34 (1.68)
Product supply issue	4	9.47 (3.55–25.26)	9.45 (30.22)	9.45 (4.16)	3.24 (1.57)
Constipation	76	8.97 (7.13–11.27)	8.72 (521.19)	8.72 (7.2)	3.12 (1.46)
Product prescribing error	13	8.78 (5.09–15.14)	8.74 (89.09)	8.73 (5.53)	3.13 (1.46)
Abdominal pain upper	63	8.13 (6.33–10.44)	7.95 (383.73)	7.95 (6.44)	2.99 (1.32)
Abdominal pain lower	7	7.91 (3.77–16.61)	7.89 (42.1)	7.89 (4.24)	2.98 (1.31)
Oral discomfort	4	7.72 (2.90–20.6)	7.71 (23.36)	7.71 (3.39)	2.95 (1.28)
Blood sodium decreased	5	7.68 (3.19–18.46)	7.66 (28.96)	7.66 (3.67)	2.94 (1.27)
Abdominal pain	60	7.09 (5.49–9.16)	6.94 (306.19)	6.94 (5.6)	2.8 (1.13)
Therapy interrupted	25	7.08 (4.77–10.49)	7.01 (129.05)	7.01 (5.04)	2.81 (1.14)
Haemorrhoids	5	6.95 (2.89–16.73)	6.94 (25.42)	6.94 (3.33)	2.79 (1.13)
Muscle spasms	47	6.90 (5.17–9.21)	6.79 (232.46)	6.78 (5.33)	2.76 (1.10)
Product quality issue	28	6.86 (4.72–9.95)	6.79 (138.44)	6.79 (4.97)	2.76 (1.10)
Sinus congestion	3	6.35 (2.04–19.7)	6.34 (13.49)	6.34 (2.46)	2.66 (1.00)
Therapeutic product effect incomplete	28	6.19 (4.27–8.99)	6.14 (120.54)	6.13 (4.49)	2.62 (0.95)
Abdominal discomfort	39	5.31 (3.87–7.28)	5.24 (134.12)	5.24 (4.02)	2.39 (0.72)
Ill-defined disorder	16	4.94 (3.02–8.08)	4.92 (49.98)	4.92 (3.26)	2.3 (0.63)
Respiratory disorder	5	4.44 (1.85–10.67)	4.43 (13.29)	4.43 (2.13)	2.15 (0.48)

PT, preferred term; ROR, reporting odds ratio; PRR, proportional reporting ratio; EBGM, empirical Bayesian geometric mean; EBGM05, the lower limit of the 95% CI, of EBGM; IC, information component; IC025, the lower limit of the 95% CI, of the IC; CI, confidence interval.

### 3.4 Subgroup analysis

Subgroup analysis was further conducted to explore adverse events with positive signals across various populations, including age and sex. Regardless of age or sex, the most frequent adverse event at PT levels was diarrhoea (Supplementary Tables S4–S7). Other common adverse events among males included drug ineffective, therapeutic product effect incomplete, constipation, abdominal pain upper, product quality issue, and flatulence (Supplementary Table S4). Constipation, drug ineffective, abdominal distension, abdominal pain upper, abdominal pain were common adverse events among female patients (Supplementary Table S5). In patients aged <65 years, frequent adverse events comprised constipation, abdominal pain upper, therapy interrupted, product quality issue, abdominal distension (Supplementary Table S6). In patients aged ≥65 years, common events included constipation, drug ineffective, therapeutic product effect incomplete, abdominal distension, abdominal pain upper, and flatulence (Supplementary Table S7).

### 3.5 Time to onset and weibull distribution analysis of adverse events

**FIGURE 3:**
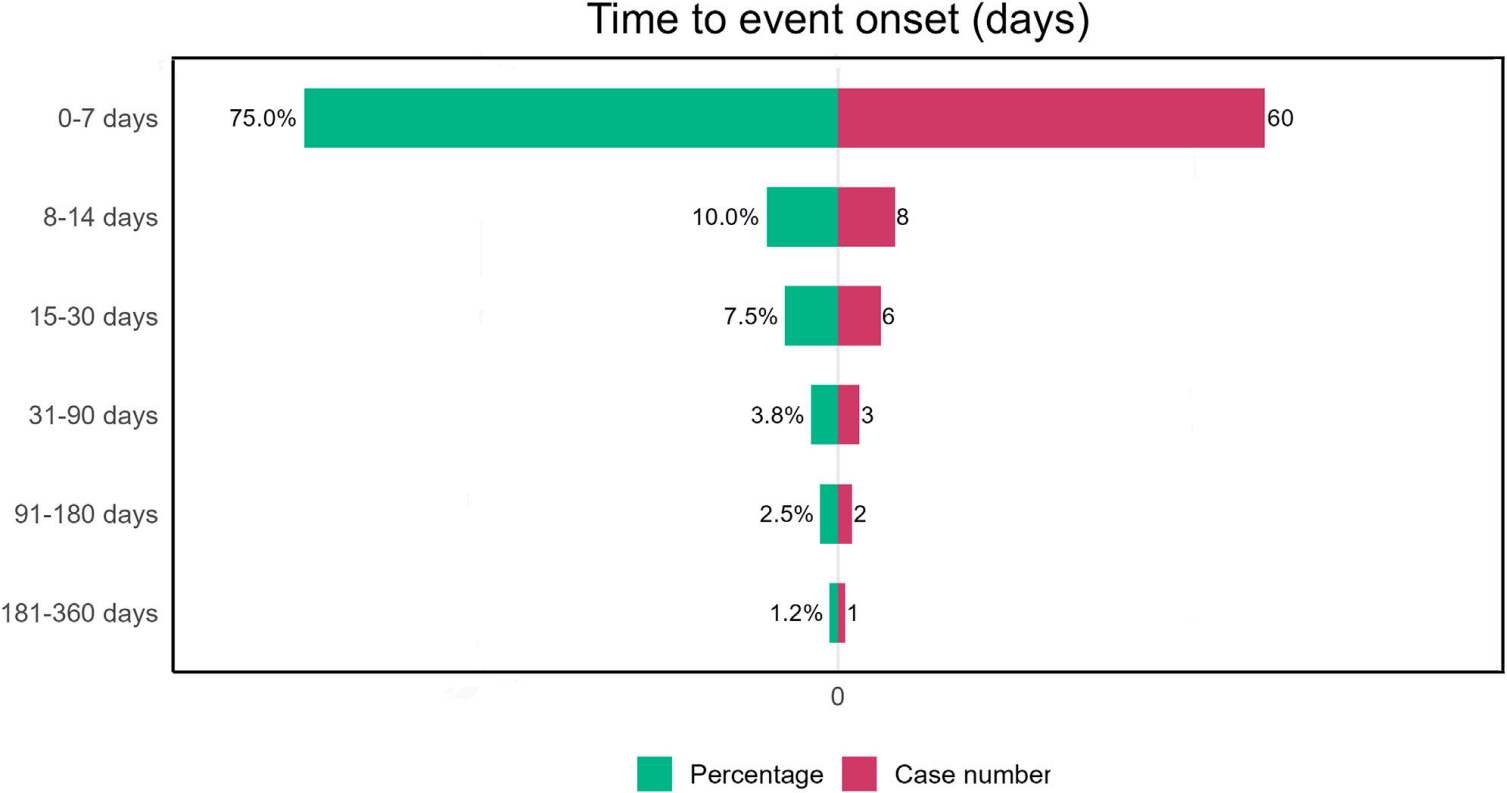
Plecanatide-induced adverse events: time to onset analysis.

**FIGURE 4:**
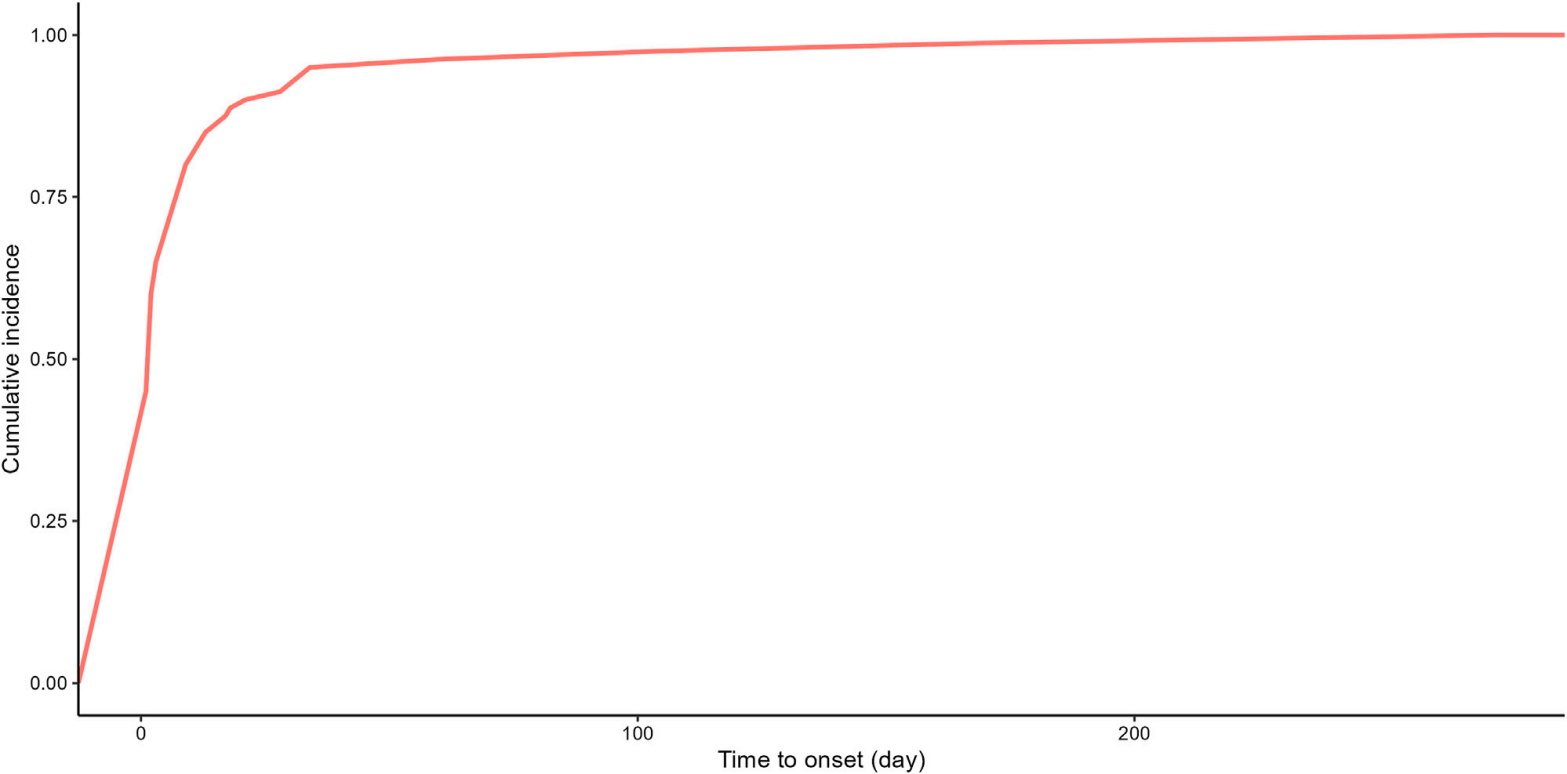
Plecanatide-related adverse events: cumulative incidence over time.

Due to the absence of onset time records for some adverse event reports, we analyzed the time to onset for 80 reports. Time to onset of plecanatide-related adverse events is shown in Figure 3. The majority of adverse events, accounting for 75%, occurred within 7 days, while only 1.2% occurred more than 180 days after initiation. Figure 4 demonstrated the cumulative incidence of adverse events associated with plecanatide over time. The median occurrence time of adverse events was 2 days. Weibull distribution analysis suggested an early failure model. Detailed parameters were displayed in Table 4.

**TABLE 4:** Time to onset of plecanatide-associated adverse events and Weibull distribution analysis.

Drug	TTO (days)		Weibull distribution		
	Case reports	Median (IQR)	Scale parameter: α (95% CI)	Shape parameter: β (95% CI)	Type
Plecanatide	80	2.00 (1.00–7.25)	6.53 (3.96–9.11)	0.59 (0.51–0.68)	Early failure

TTO, time to onset; CI, confidence interval; IQR, interquartile range.

### 3.6 Sensitivity analysis

Sensitivity analysis was conducted to further enhance the robustness of findings. The top 3 co-administered medications with pelcanatide were miralax, omeprazole, linzess, synthroid. The top 5 co-administered medications with plecanatide were miralax, omeprazole, linzess, synthroid, and lisinopril. Positive adverse events in the sensitivity analyses were consistent with those in the original disproportionality analysis. After excluding the adverse events linked to top 3 co-administered medications with plecanatide, common adverse events with positive signals at PT levels included diarrhoea, abdominal distension, constipation, abdominal pain upper, abdominal pain (Supplementary Table S8). Similarly, common adverse events with positive signals incorporated diarrhoea, constipation, abdominal distension, abdominal pain upper, and abdominal pain, with adverse events linked to top 5 co-administered medications with plecanatide excluded (Supplementary Table S9).

## 4 Discussion

This study provides a comprehensive assessment of adverse events associated with plecanatide since its market introduction in 2017. Diarrhea is identified as the most prevalent adverse event. Furthermore, several adverse events not listed in the package insert, including bloating, abdominal pain, hyponatremia, respiratory disorders, thirst, oral discomfort, sinus headache, sinusitis, and muscle cramps, were also identified, highlighting the importance of drug monitoring.

During the 12-week treatment period for IBS-C, diarrhea was the most frequently reported adverse event, occurring in 4.3% of patients receiving 3 mg plecanatide and 4.0% of those receiving 6 mg plecanatide (Brenner et al., 2023). In the 12-week treatment period for CIC, diarrhea was also the most common adverse event, with rates of 1.3% for placebo, 5.9% for 3 mg, and 5.7% for 6 mg (Miner et al., 2017). These findings align with the results of our study, where diarrhea was the most frequently reported adverse event, comprising 275 cases. Given both past and current research, it is crucial to closely monitor plecanatide’s adverse events and remain vigilant about the occurrence of diarrhea.

In the disproportionality analysis, we first examined plecanatide’s adverse events across the SOC hierarchy, identifying gastrointestinal disorders as the positive signal with the highest strength. This is closely linked to its primary indications and mechanism of action. The SOC level provides a broad overview of adverse events, helping to identify the systems most affected (Jiang et al., 2024). To explore specific adverse events in more detail, we further conducted a disproportionality analysis at the PT level. Adverse events with high signal strength include patient dissatisfaction with treatment, rectal tenesmus, increased fecal volume, abnormal gastrointestinal sounds, gastrointestinal motility disorder, dyschezia, bowel movement irregularity, hard stools, inability to afford medication, and defecation urgency. With the exception of dissatisfaction with treatment efficacy and cost, all these adverse events were related to gastrointestinal disorders.

Rectal tenesmus is a classic gastrointestinal symptom, potentially caused by inflammatory stimulation of the intestinal mucosa and increased mucus secretion (Gajendran et al., 2019). Plecanatide and its active metabolites bind to and activate the catalytic domain of GC-C on the intestinal epithelial surface. This activation leads to increased production of cGMP, which in turn activates the cystic fibrosis transmembrane conductance regulator, stimulating chloride and bicarbonate secretion. As a result, intestinal fluid increases, accelerating intestinal transit (Pitari, 2013; Shailubhai et al., 2013). This mechanism can trigger or worsen rectal tenesmus and also contributes to increased stool volume. Diarrhea, characterized by loose stools as well as an increase in stool frequency, weight, or volume, may occur (Sokic-Milutinovic et al., 2022). When stool volume rises, clinicians should be vigilant for potential diarrhea.

Gastrointestinal motility disorder and dyschezia are notable adverse events with high signal strength. These events are commonly seen in patients with CIC or IBS-C, which can complicate distinguishing between primary disease symptoms and drug-induced reactions when new medications are introduced. The FAERS database includes reports of adverse events from multiple sources, such as healthcare professionals, consumers, and manufacturers, which may contribute to varying interpretations of these events (Harpaz et al., 2013). Therefore, clinicians should remain especially vigilant and closely monitor for these adverse events in patients with pre-existing gastrointestinal conditions.

Beyond the aforementioned positive adverse events with high strength, we also identified several noteworthy adverse events with elevated frequencies. Notably, Nausea emerged as a common adverse event. Nausea can reduce appetite, thereby affecting quality of life, medication adherence, and overall therapeutic efficacy, which may impact disease control and diminish patient wellbeing. The causes of nausea are multifactorial, involving gastrointestinal conditions such as gastroparesis and cyclic vomiting syndrome, as well as non-gastrointestinal conditions like vestibular and neurological disorders (Lacy et al., 2018). However, the mechanism by which plecanatide induces nausea remains unclear and requires further research to elucidate its pathophysiology.

Hyponatremia is another notable adverse effect, characterized by a serum sodium concentration below 136 mmol/L. It is commonly associated with conditions like hypovolemia, liver cirrhosis, heart failure, or inappropriate medication use (Gross, 2008). Given plecanatide’s mechanism, which involves increased intestinal fluid production and accelerated transit, it is plausible that plecanatide could contribute to hyponatremia. Non-pharmacological strategies for managing constipation often include increased fluid intake, which may exacerbate dilutional hyponatremia. While standard treatment for hyponatremia involves fluid restriction, this may worsen constipation symptoms. Mild to moderate hyponatremia is associated with cognitive impairment and can be life-threatening in severe cases (Gross, 2008; Gendreau and Potenza, 2014). Clinicians must closely monitor electrolyte levels when administering plecanatide to prevent hyponatremia.

Subgroup analysis revealed that anal incontinence and negative thoughts are more common among male patients, while hypogeusia and colitis are exclusively observed in male patients. Clinicians should exercise caution when managing these patients. For female patients, attention should be paid to muscle cramps, chills, and palpitations. Although plecanatide has been well-tolerated in patients aged 65 and older with CIC or IBS-C, there is limited data on its safety in the elderly (Menees et al., 2020). The subgroup analysis underscores the need for vigilance regarding muscle cramps and weight loss in elderly patients. Sensitivity analyses, which included altering concomitant medications, showed no significant impact on plecanatide’s adverse effects. Diarrhea remains the most common adverse effect across all populations, with abdominal pain, bloating, and constipation also frequently reported. Additionally, patients of all genders and ages have expressed concerns about the inability to afford medication. The cost of medication is a significant barrier to effective treatment adherence, suggesting that clinical pharmacists should consider drug prices when selecting medications to improve patient adherence (Clark, 1991). These non-lethal but notable adverse events may affect patient adherence and, consequently, therapeutic outcomes. Thus, addressing these specific adverse events is crucial for improving treatment results and enhancing drug efficacy.

Time analysis shows that plecanatide-related adverse events tend to occur within 7.25 days, with Weibull analysis clustering these events around 6.53 days. This suggests that adverse events are more likely to arise soon after starting the medication due to drug intolerance or reactions. Clinicians should focus on detecting and managing adverse events during the first week of treatment to ensure patient safety and adherence.

This study has several strengths. It leverages the FAERS database to investigate plecanatide’s adverse events in real-world settings, enabling the early detection of new safety signals post-market and providing valuable guidance for clinicians. Moreover, the study employs sophisticated analytical methods, including disproportionality analysis, subgroup analysis, sensitivity analysis, and event analysis, offering a comprehensive understanding of plecanatide’s adverse events in the population.

However, this study has limitations. The FAERS database has inherent flaws, including underreporting of adverse events and overreporting of events unrelated to the medication. The quality and accuracy of reports depend on the expertise of the reporters (Stephenson and Hauben, 2007). Additionally, the thresholds used in the study need further investigation, as varying thresholds may affect the results. Finally, the limited and regionally concentrated data necessitate broader, long-term studies to validate these findings.

## 5 Conclusion

This study provides a preliminary assessment of the safety of plecanatide in real-world applications, revealing significant new adverse event signals. These findings will provide insights into plecanatide’s safety profile, supporting clinicians in making more informed prescribing decisions for patients with CIC and IBS-C. Future research should further validate these signals and systematically evaluate the long-term safety of plecanatide.

## Data Availability

The datasets presented in this study can be found in online repositories. The names of the repository/repositories and accession number(s) can be found below: https://fis.fda.gov/extensions/FPD-QDE-FAERS/FPD-QDE-FAERS.html.
