# Supplementary material for: Assessing real-world safety of plecanatide: a pharmacovigilance study based on the FDA adverse event reporting system
Source: Front Pharmacol. 2024 Nov 25;15:1500810. doi: 10.3389/fphar.2024.1500810 (PMC11625541; doi:10.3389/fphar.2024.1500810)
Supplement: Supplementary file 1 [file Table1.DOCX]

**Supplementary Materials**

This appendix has been provided by the authors to give readers additional information about their work.

**List of Contents**

Page 1: Cover page

Page 2: List of contents

Page 3: Supplementary Table S1

Page 4: Supplementary Table S2

Page 5-8: Supplementary Table S3

Page 9-10: Supplementary Table S4

Page 11-13: Supplementary Table S5

Page 14-15: Supplementary Table S6

Page 16-17: Supplementary Table S7

Page 18-21: Supplementary Table S8

Page 22-25: Supplementary Table S9

**Supplementary Table S1.** Two-by-two contingency table for disproportionality analyses.

|  | Target AEs | Other AEs | Total |
| --- | --- | --- | --- |
| Plecanatide | a | b | a+b |
| Other drugs | c | d | c+d |
| Total | a+c | b+d | a+b+c+d |

AEs, adverse events; a, number of reports containing both the target drug and target adverse drug reaction; b, number of reports containing other adverse drug reaction of the target drug; c, number of reports containing the target adverse drug reaction of other drugs; d, number of reports containing other drugs and other adverse drug reactions.

**Supplementary Table S2.** Four major algorithms used for signal detection.

| Algorithms | Equation | Criteria |
| --- | --- | --- |
| ROR | ROR=ad/b/c | lower limit of 95% CI>1, N≥3 |
|  | 95%CI=e^ln(ROR)±1.96(1/a+1/b+1/c+1/d)^0.5^ |  |
| PRR | PRR=a(c+d)/c/(a+b) | PRR≥2, χ^2^≥4, N≥3 |
|  | χ^2^=[(ad-bc)^2](a+b+c+d)/[(a+b)(c+d)(a+c)(b+d)] |  |
| BCPNN | IC=log_2_a(a+b+c+d)(a+c)(a+b) | IC025>0 |
|  | 95%CI= E(IC) ± 2V(IC)^0.5 |  |
| MGPS | EBGM=a(a+b+c+d)/(a+c)/(a+b) | EBGM05>2 |
|  | 95%CI=e^ln(EBGM)±1.96(1/a+1/b+1/c+1/d)^0.5^ |  |

a, number of reports containing both the target drug and target adverse drug reaction; b, number of reports containing other adverse drug reaction of the target drug; c, number of reports containing the target adverse drug reaction of other drugs; d, number of reports containing other drugs and other adverse drug reactions. 95%CI, 95% confidence interval; N, the number of reports; χ2, chi-squared; IC, information component; IC025, the lower limit of 95% CI of the IC; E(IC), the IC expectations; V(IC), the variance of IC; EBGM, empirical Bayesian geometric mean; EBGM05, the lower limit of 95% CI of EBGM.

**Supplementary Table S3.** All adverse events meeting the positive signal threshold at the PT levels.

| PT | Case reports | ROR (95% Cl) | PRR (χ^2^) | EBGM (EBGM05) | IC (IC025) |
| --- | --- | --- | --- | --- | --- |
| Diarrhoea | 275 | 11.16 (9.84 - 12.64) | 10.04 (2261.73) | 10.03 (9.04) | 3.33 (1.66) |
| Abdominal distension | 77 | 20.5 (16.33 - 25.72) | 19.9 (1381.91) | 19.87 (16.43) | 4.31 (2.65) |
| Constipation | 76 | 8.97 (7.13 - 11.27) | 8.72 (521.19) | 8.72 (7.2) | 3.12 (1.46) |
| Abdominal pain upper | 63 | 8.13 (6.33 - 10.44) | 7.95 (383.73) | 7.95 (6.44) | 2.99 (1.32) |
| Nausea | 60 | 2.02 (1.57 - 2.61) | 2 (30.25) | 3.54 (2.23) | 2.89 (1.15) |
| Abdominal pain | 60 | 7.09 (5.49 - 9.16) | 6.94 (306.19) | 6.94 (5.6) | 2.80 (1.13) |
| Flatulence | 53 | 26.08 (19.86 - 34.24) | 25.55 (1248.51) | 25.5 (20.3) | 4.67 (3.01) |
| Muscle spasms | 47 | 6.90 (5.17 - 9.21) | 6.79 (232.46) | 6.78 (5.33) | 2.76 (1.10) |
| Abdominal discomfort | 39 | 5.31 (3.87 - 7.28) | 5.24 (134.12) | 5.24 (4.02) | 2.39 (0.72) |
| Therapeutic product effect decreased | 30 | 10.71 (7.47 - 15.35) | 10.59 (260.59) | 10.58 (7.83) | 3.4 (1.74) |
| Product quality issue | 28 | 6.86 (4.72 - 9.95) | 6.79 (138.44) | 6.79 (4.97) | 2.76 (1.1) |
| Therapeutic product effect incomplete | 28 | 6.19 (4.27 - 8.99) | 6.14 (120.54) | 6.13 (4.49) | 2.62 (0.95) |
| Product use issue | 26 | 3.04 (2.07 - 4.47) | 3.02 (35.21) | 3.02 (2.18) | 1.59 (-0.07) |
| Therapy interrupted | 25 | 7.08 (4.77 - 10.49) | 7.01 (129.05) | 7.01 (5.04) | 2.81 (1.14) |
| Patient dissatisfaction with treatment | 23 | 298.25 (196.89 - 451.8) | 295.52 (6597.08) | 288.79 (204.02) | 8.17 (6.5) |
| Inability to afford medication | 20 | 38.5 (24.78 - 59.82) | 38.2 (722.54) | 38.09 (26.34) | 5.25 (3.58) |
| Dehydration | 17 | 3.73 (2.32 - 6.02) | 3.71 (33.78) | 3.71 (2.49) | 1.89 (0.23) |
| Frequent bowel movements | 17 | 14.43 (8.95 - 23.25) | 14.34 (210.78) | 14.32 (9.61) | 3.84 (2.17) |
| Bowel movement irregularity | 16 | 45.98 (28.1 - 75.24) | 45.69 (697.04) | 45.53 (30.16) | 5.51 (3.84) |
| Ill-defined disorder | 16 | 4.94 (3.02 - 8.08) | 4.92 (49.98) | 4.92 (3.26) | 2.30 (0.63) |
| Faeces discoloured | 16 | 21.12 (12.91 - 34.53) | 20.99 (304.14) | 20.95 (13.88) | 4.39 (2.72) |
| Gastrointestinal sounds abnormal | 15 | 79.62 (47.85 - 132.48) | 79.15 (1150.35) | 78.67 (51.38) | 6.3 (4.63) |
| Product prescribing error | 13 | 8.78 (5.09 - 15.14) | 8.74 (89.09) | 8.73 (5.53) | 3.13 (1.46) |
| Gastrointestinal motility disorder | 13 | 58.11 (33.65 - 100.34) | 57.81 (722.55) | 57.55 (36.44) | 5.85 (4.18) |
| Insurance issue | 12 | 17.4 (9.86 - 30.69) | 17.32 (184.35) | 17.3 (10.76) | 4.11 (2.45) |
| Defaecation urgency | 11 | 37.82 (20.9 - 68.44) | 37.66 (391.42) | 37.55 (22.86) | 5.23 (3.56) |
| Dry mouth | 10 | 3.47 (1.87 - 6.46) | 3.46 (17.52) | 3.46 (2.06) | 1.79 (0.12) |
| Gastrointestinal pain | 10 | 22.15 (11.89 - 41.23) | 22.06 (200.75) | 22.02 (13.09) | 4.46 (2.79) |
| Anal incontinence | 10 | 20.31 (10.91 - 37.81) | 20.23 (182.53) | 20.2 (12.01) | 4.34 (2.67) |
| Dyschezia | 9 | 52.31 (27.15 - 100.79) | 52.12 (449.47) | 51.91 (29.99) | 5.70 (4.03) |
| Rectal tenesmus | 9 | 194.54 (100.6 - 376.21) | 193.85 (1700.63) | 190.94 (109.96) | 7.58 (5.91) |
| Sleep disorder | 8 | 2.71 (1.35 - 5.42) | 2.70 (8.57) | 2.7 (1.51) | 1.43 (-0.23) |
| Chest discomfort | 8 | 2.05 (1.03 - 4.11) | 2.05 (4.32) | 2.05 (1.15) | 1.04 (-0.63) |
| Abdominal pain lower | 7 | 7.91 (3.77 - 16.61) | 7.89 (42.1) | 7.89 (4.24) | 2.98 (1.31) |
| Faeces hard | 7 | 42.48 (20.21 - 89.31) | 42.37 (281.8) | 42.23 (22.68) | 5.40 (3.73) |
| Symptom recurrence | 7 | 21.26 (10.12 - 44.67) | 21.2 (134.54) | 21.17 (11.37) | 4.40 (2.74) |
| Thirst | 7 | 10.19 (4.85 - 21.4) | 10.16 (57.81) | 10.16 (5.46) | 3.34 (1.68) |
| Abdominal tenderness | 6 | 36.43 (16.33 - 81.27) | 36.35 (205.68) | 36.25 (18.52) | 5.18 (3.51) |
| Blood sodium decreased | 5 | 7.68 (3.19 - 18.46) | 7.66 (28.96) | 7.66 (3.67) | 2.94 (1.27) |
| Haemorrhoids | 5 | 6.95 (2.89 - 16.73) | 6.94 (25.42) | 6.94 (3.33) | 2.79 (1.13) |
| Respiratory disorder | 5 | 4.44 (1.85 - 10.67) | 4.43 (13.29) | 4.43 (2.13) | 2.15 (0.48) |
| Product packaging difficult to open | 5 | 37.77 (15.69 - 90.94) | 37.70 (178.09) | 37.59 (18.02) | 5.23 (3.56) |
| Haemorrhoidal haemorrhage | 5 | 25.42 (10.56 - 61.17) | 25.37 (116.81) | 25.32 (12.14) | 4.66 (2.99) |
| Proctalgia | 5 | 21.34 (8.87 - 51.35) | 21.3 (96.58) | 21.26 (10.2) | 4.41 (2.74) |
| Abnormal faeces | 5 | 14.4 (5.99 - 34.65) | 14.38 (62.17) | 14.36 (6.89) | 3.84 (2.18) |
| Product supply issue | 4 | 9.47 (3.55 - 25.26) | 9.45 (30.22) | 9.45 (4.16) | 3.24 (1.57) |
| Oral discomfort | 4 | 7.72 (2.90 - 20.6) | 7.71 (23.36) | 7.71 (3.39) | 2.95 (1.28) |
| Faecaloma | 4 | 19.74 (7.4 - 52.67) | 19.71 (70.93) | 19.68 (8.66) | 4.3 (2.63) |
| Faecal volume increased | 4 | 185.54 (69.09 - 498.31) | 185.25 (722.43) | 182.59 (79.89) | 7.51 (5.84) |
| Product solubility abnormal | 4 | 16.11 (6.04 – 43.00) | 16.09 (56.54) | 16.07 (7.07) | 4.01 (2.34) |
| Sinus congestion | 3 | 6.35 (2.04 - 19.7) | 6.34 (13.49) | 6.34 (2.46) | 2.66 (1.00) |
| Anorectal discomfort | 3 | 22.94 (7.39 - 71.26) | 22.92 (62.77) | 22.88 (8.86) | 4.52 (2.85) |
| Sinus headache | 3 | 19.35 (6.23 - 60.1) | 19.33 (52.07) | 19.3 (7.48) | 4.27 (2.6) |
| Functional gastrointestinal disorder | 3 | 19.34 (6.23 - 60.07) | 19.32 (52.05) | 19.29 (7.48) | 4.27 (2.6) |
| Therapeutic product effect variable | 3 | 12.88 (4.15 - 40) | 12.87 (32.81) | 12.86 (4.98) | 3.68 (2.02) |
| Polyp | 3 | 10.91 (3.51 - 33.86) | 10.9 (26.94) | 10.89 (4.22) | 3.44 (1.78) |

PT, preferred term; ROR, reporting odds ratio; PRR, proportional reporting ratio; EBGM, empirical Bayesian geometric mean; EBGM05, the lower limit of the 95% CI of EBGM; IC, information component; IC025, the lower limit of the 95% CI of the IC; CI, confidence interval.

**Supplementary Table S4.** All adverse events meeting the positive signal threshold at the PT levels in males.

| PT | Case reports | ROR (95% Cl) | PRR (χ^2^) | EBGM (EBGM05) | IC (IC025) |
| --- | --- | --- | --- | --- | --- |
| Diarrhoea | 45 | 12.85 (9.41 - 17.56) | 11.41 (431.72) | 11.4 (8.78) | 3.51 (1.84) |
| Therapeutic product effect incomplete | 13 | 21.7 (12.47 - 37.74) | 20.97 (247.43) | 20.95 (13.18) | 4.39 (2.72) |
| Constipation | 12 | 9.8 (5.52 - 17.43) | 9.52 (91.76) | 9.52 (5.88) | 3.25 (1.58) |
| Abdominal pain upper | 11 | 11.82 (6.49 - 21.53) | 11.5 (105.63) | 11.49 (6.95) | 3.52 (1.85) |
| Product quality issue | 10 | 21.35 (11.39 - 40.04) | 20.8 (188.6) | 20.79 (12.28) | 4.38 (2.7) |
| Flatulence | 10 | 32.17 (17.15 - 60.32) | 31.32 (293.49) | 31.29 (18.49) | 4.97 (3.29) |
| Therapeutic product effect decreased | 9 | 28.73 (14.82 - 55.68) | 28.05 (234.77) | 28.03 (16.11) | 4.81 (3.14) |
| Abdominal distension | 7 | 13.97 (6.61 - 29.52) | 13.72 (82.65) | 13.72 (7.34) | 3.78 (2.11) |
| Patient dissatisfaction with treatment | 6 | 733.44 (324.1 - 1659.77) | 721.53 (4211.11) | 703.81 (355.37) | 9.46 (7.78) |
| Inappropriate schedule of product administration | 6 | 4.18 (1.87 - 9.37) | 4.13 (14.28) | 4.13 (2.1) | 2.05 (0.37) |
| Faeces discoloured | 6 | 46.46 (20.72 - 104.16) | 45.72 (262.12) | 45.65 (23.23) | 5.51 (3.84) |
| Therapy interrupted | 5 | 9.64 (3.99 - 23.3) | 9.52 (38.18) | 9.52 (4.55) | 3.25 (1.58) |
| Inability to afford medication | 5 | 67.4 (27.86 - 163.07) | 66.5 (321.88) | 66.35 (31.68) | 6.05 (4.38) |
| Gastrointestinal motility disorder | 5 | 156.99 (64.8 - 380.34) | 154.87 (760.35) | 154.04 (73.47) | 7.27 (5.59) |
| Defaecation urgency | 5 | 100.45 (41.5 - 243.15) | 99.1 (483.95) | 98.76 (47.13) | 6.63 (4.95) |
| Anal incontinence | 5 | 71.31 (29.47 - 172.54) | 70.36 (341.08) | 70.19 (33.51) | 6.13 (4.46) |
| Frequent bowel movements | 4 | 19.86 (7.41 - 53.22) | 19.66 (70.82) | 19.64 (8.61) | 4.3 (2.62) |
| Product prescribing error | 3 | 12.72 (4.08 - 39.63) | 12.62 (32.11) | 12.62 (4.88) | 3.66 (1.98) |
| Contusion | 3 | 7.19 (2.31 - 22.39) | 7.14 (15.85) | 7.14 (2.76) | 2.84 (1.16) |

PT, preferred term; ROR, reporting odds ratio; PRR, proportional reporting ratio; EBGM, empirical Bayesian geometric mean; EBGM05, the lower limit of the 95% CI of EBGM; IC, information component; IC025, the lower limit of the 95% CI of the IC; CI, confidence interval.

**Supplementary Table S5.** All adverse events meeting the positive signal threshold at the PT levels in females.

| PT | Case reports | ROR (95% Cl) | PRR (χ^2^) | EBGM (EBGM05) | IC (IC025) |
| --- | --- | --- | --- | --- | --- |
| Diarrhoea | 119 | 9.33 (7.72 - 11.27) | 8.51 (797.32) | 8.5 (7.26) | 3.09 (1.42) |
| Constipation | 45 | 10.87 (8.07 - 14.65) | 10.51 (388.16) | 10.5 (8.18) | 3.39 (1.72) |
| Abdominal distension | 34 | 16.89 (12.01 - 23.76) | 16.45 (493.51) | 16.43 (12.35) | 4.04 (2.37) |
| Abdominal pain upper | 26 | 6 (4.07 - 8.86) | 5.9 (106.05) | 5.89 (4.26) | 2.56 (0.89) |
| Abdominal pain | 23 | 5.21 (3.45 - 7.87) | 5.13 (76.68) | 5.13 (3.63) | 2.36 (0.69) |
| Therapy interrupted | 20 | 10.53 (6.77 - 16.38) | 10.37 (169.47) | 10.36 (7.16) | 3.37 (1.71) |
| Therapeutic product effect decreased | 19 | 13.57 (8.62 - 21.35) | 13.37 (217.54) | 13.36 (9.14) | 3.74 (2.07) |
| Product quality issue | 18 | 8.57 (5.38 - 13.65) | 8.46 (118.5) | 8.45 (5.73) | 3.08 (1.41) |
| Flatulence | 17 | 16.81 (10.41 - 27.15) | 16.59 (249.01) | 16.57 (11.1) | 4.05 (2.38) |
| Patient dissatisfaction with treatment | 17 | 444.8 (273.62 - 723.07) | 438.55 (7202.26) | 425.62 (283.43) | 8.73 (7.06) |
| Therapeutic product effect incomplete | 15 | 7.18 (4.31 - 11.95) | 7.1 (78.77) | 7.1 (4.64) | 2.83 (1.16) |
| Muscle spasms | 14 | 3.74 (2.21 - 6.34) | 3.71 (27.82) | 3.71 (2.39) | 1.89 (0.22) |
| Abdominal discomfort | 14 | 3.49 (2.06 - 5.9) | 3.46 (24.52) | 3.46 (2.22) | 1.79 (0.12) |
| Inability to afford medication | 14 | 55.24 (32.59 - 93.66) | 54.61 (734.23) | 54.41 (34.98) | 5.77 (4.1) |
| Ill-defined disorder | 12 | 11.43 (6.47 - 20.19) | 11.33 (113.01) | 11.32 (7.03) | 3.5 (1.83) |
| Dehydration | 11 | 5.03 (2.78 - 9.11) | 5 (35.21) | 4.99 (3.04) | 2.32 (0.65) |
| Bowel movement irregularity | 10 | 62.77 (33.64 - 117.13) | 62.26 (600.26) | 62 (36.79) | 5.95 (4.29) |
| Insurance issue | 10 | 27.71 (14.86 - 51.66) | 27.49 (254.82) | 27.44 (16.29) | 4.78 (3.11) |
| Product prescribing error | 8 | 11.94 (5.96 - 23.94) | 11.87 (79.6) | 11.86 (6.63) | 3.57 (1.9) |
| Frequent bowel movements | 7 | 12.66 (6.02 - 26.62) | 12.59 (74.66) | 12.58 (6.75) | 3.65 (1.98) |
| Therapy cessation | 7 | 4.78 (2.27 - 10.05) | 4.76 (20.79) | 4.76 (2.55) | 2.25 (0.58) |
| Gastrointestinal motility disorder | 6 | 51.51 (23.06 - 115.05) | 51.26 (294.64) | 51.08 (26.08) | 5.67 (4.01) |
| Symptom recurrence | 6 | 40.22 (18.01 - 89.8) | 40.02 (227.68) | 39.91 (20.38) | 5.32 (3.65) |
| Dyschezia | 5 | 62.95 (26.1 - 151.81) | 62.7 (302.26) | 62.43 (29.89) | 5.96 (4.29) |
| Gastrointestinal sounds abnormal | 5 | 53.57 (22.22 - 129.15) | 53.35 (255.93) | 53.16 (25.46) | 5.73 (4.06) |
| Product availability issue | 5 | 9.4 (3.9 - 22.64) | 9.37 (37.36) | 9.36 (4.49) | 3.23 (1.56) |
| Defaecation urgency | 4 | 30.43 (11.39 - 81.31) | 30.34 (113.25) | 30.28 (13.3) | 4.92 (3.25) |
| Faeces discoloured | 4 | 11.72 (4.39 - 31.29) | 11.68 (39.06) | 11.68 (5.13) | 3.55 (1.88) |
| Product supply issue | 4 | 20.03 (7.5 - 53.49) | 19.97 (71.97) | 19.94 (8.76) | 4.32 (2.65) |
| Expired product administered | 4 | 4.9 (1.84 - 13.08) | 4.89 (12.37) | 4.89 (2.15) | 2.29 (0.62) |
| Extra dose administered | 4 | 5.04 (1.89 - 13.44) | 5.02 (12.89) | 5.02 (2.21) | 2.33 (0.66) |
| Gastrointestinal pain | 4 | 16.34 (6.12 - 43.64) | 16.29 (57.36) | 16.28 (7.16) | 4.02 (2.36) |
| Product solubility abnormal | 4 | 34.53 (12.92 - 92.26) | 34.42 (129.49) | 34.34 (15.09) | 5.1 (3.43) |
| Abdominal pain lower | 3 | 5.26 (1.7 - 16.35) | 5.25 (10.33) | 5.25 (2.04) | 2.39 (0.72) |
| Thirst | 3 | 8 (2.58 - 24.86) | 7.99 (18.33) | 7.98 (3.09) | 3 (1.33) |
| Head injury | 3 | 5.35 (1.72 - 16.61) | 5.34 (10.58) | 5.34 (2.07) | 2.42 (0.75) |
| Product packaging difficult to open | 3 | 42.36 (13.62 - 131.74) | 42.25 (120.49) | 42.13 (16.3) | 5.4 (3.73) |
| Anal incontinence | 3 | 11.79 (3.8 - 36.63) | 11.76 (29.53) | 11.75 (4.55) | 3.56 (1.89) |
| Respiratory disorder | 3 | 5.57 (1.79 - 17.29) | 5.56 (11.21) | 5.55 (2.15) | 2.47 (0.81) |
| Abnormal faeces | 3 | 18.99 (6.11 - 59.02) | 18.95 (50.94) | 18.93 (7.33) | 4.24 (2.57) |

PT, preferred term; ROR, reporting odds ratio; PRR, proportional reporting ratio; EBGM, empirical Bayesian geometric mean; EBGM05, the lower limit of the 95% CI of EBGM; IC, information component; IC025, the lower limit of the 95% CI of the IC; CI, confidence interval.

**Supplementary Table S6.** All adverse events meeting the positive signal threshold at the PT levels in individuals aged < 65 years.

| PT | Case reports | ROR (95% Cl) | PRR (χ^2^) | EBGM (EBGM05) | IC (IC025) |
| --- | --- | --- | --- | --- | --- |
| Diarrhoea | 39 | 8.98 (6.47 - 12.46) | 8.31 (253.41) | 8.31 (6.32) | 3.06 (1.38) |
| Constipation | 20 | 16.53 (10.56 - 25.88) | 15.87 (279.29) | 15.86 (10.9) | 3.99 (2.32) |
| Abdominal pain upper | 13 | 8.37 (4.82 - 14.53) | 8.17 (82.03) | 8.17 (5.15) | 3.03 (1.36) |
| Therapy interrupted | 13 | 19.63 (11.31 - 34.07) | 19.11 (223.31) | 19.1 (12.04) | 4.26 (2.58) |
| Product quality issue | 13 | 23.57 (13.58 - 40.91) | 22.94 (272.91) | 22.92 (14.45) | 4.52 (2.85) |
| Abdominal distension | 11 | 14.55 (8 - 26.47) | 14.24 (135.52) | 14.23 (8.63) | 3.83 (2.16) |
| Inability to afford medication | 9 | 152.01 (78.45 - 294.54) | 149.12 (1316.87) | 148.29 (85.26) | 7.21 (5.54) |
| Therapeutic product effect decreased | 9 | 19.63 (10.14 - 37.97) | 19.27 (155.94) | 19.26 (11.09) | 4.27 (2.6) |
| Therapeutic product effect incomplete | 6 | 8 (3.57 - 17.9) | 7.91 (36.26) | 7.91 (4.03) | 2.98 (1.31) |
| Insurance issue | 6 | 41.89 (18.71 - 93.79) | 41.37 (236.07) | 41.31 (21.05) | 5.37 (3.7) |
| Flatulence | 5 | 15.15 (6.27 - 36.58) | 15 (65.34) | 14.99 (7.17) | 3.91 (2.23) |
| Dehydration | 4 | 5.55 (2.07 - 14.85) | 5.51 (14.78) | 5.51 (2.42) | 2.46 (0.79) |
| Heart rate increased | 4 | 5.08 (1.9 - 13.6) | 5.05 (13) | 5.05 (2.21) | 2.34 (0.66) |
| Bowel movement irregularity | 4 | 72.45 (27.04 - 194.1) | 71.84 (278.69) | 71.65 (31.41) | 6.16 (4.49) |
| Patient dissatisfaction with treatment | 4 | 368.56 (136.82 - 992.82) | 365.44 (1433.89) | 360.45 (157.31) | 8.49 (6.81) |
| Gastrooesophageal reflux disease | 3 | 6.09 (1.96 - 18.94) | 6.05 (12.67) | 6.05 (2.34) | 2.6 (0.93) |
| Rectal haemorrhage | 3 | 11.1 (3.57 - 34.56) | 11.04 (27.4) | 11.04 (4.27) | 3.46 (1.79) |
| Symptom recurrence | 3 | 49.58 (15.92 - 154.46) | 49.27 (141.63) | 49.18 (19.01) | 5.62 (3.95) |
| Product prescribing error | 3 | 11.91 (3.82 - 37.06) | 11.84 (29.76) | 11.83 (4.58) | 3.56 (1.89) |
| Ill-defined disorder | 3 | 10.6 (3.41 - 32.99) | 10.54 (25.91) | 10.53 (4.07) | 3.4 (1.73) |

PT, preferred term; ROR, reporting odds ratio; PRR, proportional reporting ratio; EBGM, empirical Bayesian geometric mean; EBGM05, the lower limit of the 95% CI of EBGM; IC, information component; IC025, the lower limit of the 95% CI of the IC; CI, confidence interval.

**Supplementary Table S7.** All adverse events meeting the positive signal threshold at the PT levels in individuals aged ≥ 65 years.

| PT | Case reports | ROR (95% Cl) | PRR (χ^2^) | EBGM (EBGM05) | IC (IC025) |
| --- | --- | --- | --- | --- | --- |
| Diarrhoea | 71 | 9.12 (7.12 - 11.68) | 8.18 (453.53) | 8.17 (6.65) | 3.03 (1.36) |
| Constipation | 19 | 6.77 (4.29 - 10.69) | 6.59 (90.49) | 6.59 (4.49) | 2.72 (1.05) |
| Therapeutic product effect incomplete | 17 | 18.47 (11.4 - 29.92) | 17.98 (272.64) | 17.96 (11.99) | 4.17 (2.5) |
| Abdominal distension | 14 | 14.81 (8.72 - 25.17) | 14.5 (176) | 14.48 (9.29) | 3.86 (2.19) |
| Abdominal pain upper | 14 | 7.25 (4.27 - 12.32) | 7.11 (73.71) | 7.11 (4.56) | 2.83 (1.16) |
| Flatulence | 14 | 21.69 (12.76 - 36.86) | 21.22 (269.49) | 21.18 (13.59) | 4.4 (2.73) |
| Patient dissatisfaction with treatment | 12 | 985.04 (543.43 - 1785.52) | 965.81 (10658.86) | 890.14 (541.16) | 9.8 (8.11) |
| Abdominal discomfort | 11 | 6.64 (3.65 - 12.05) | 6.54 (51.68) | 6.53 (3.97) | 2.71 (1.04) |
| Abdominal pain | 9 | 4.43 (2.29 - 8.55) | 4.38 (23.52) | 4.38 (2.52) | 2.13 (0.46) |
| Gastrointestinal motility disorder | 8 | 115.55 (57.32 - 232.91) | 114.05 (887.68) | 112.93 (62.82) | 6.82 (5.15) |
| Muscle spasms | 7 | 3.92 (1.86 - 8.27) | 3.89 (15.07) | 3.89 (2.08) | 1.96 (0.29) |
| Ill-defined disorder | 7 | 21.68 (10.28 - 45.7) | 21.44 (136.23) | 21.4 (11.47) | 4.42 (2.75) |
| Product quality issue | 7 | 9.25 (4.39 - 19.49) | 9.15 (50.86) | 9.15 (4.9) | 3.19 (1.52) |
| Therapy interrupted | 7 | 6.56 (3.11 - 13.83) | 6.5 (32.6) | 6.49 (3.48) | 2.7 (1.03) |
| Frequent bowel movements | 6 | 22.65 (10.13 - 50.65) | 22.43 (122.69) | 22.39 (11.42) | 4.48 (2.81) |
| Therapeutic product effect decreased | 6 | 16.19 (7.24 - 36.2) | 16.04 (84.55) | 16.02 (8.17) | 4 (2.33) |
| Inability to afford medication | 6 | 52.92 (23.64 - 118.49) | 52.42 (301.28) | 52.18 (26.59) | 5.71 (4.03) |
| Dyschezia | 5 | 102.88 (42.5 - 249.05) | 102.05 (495.89) | 101.15 (48.28) | 6.66 (4.99) |
| Intentional product use issue | 5 | 4.28 (1.78 - 10.33) | 4.26 (12.47) | 4.25 (2.04) | 2.09 (0.42) |
| Product packaging difficult to open | 4 | 105.76 (39.39 - 283.97) | 105.08 (408.59) | 104.12 (45.56) | 6.7 (5.03) |
| Oropharyngeal pain | 4 | 4.68 (1.75 - 12.52) | 4.66 (11.5) | 4.66 (2.04) | 2.22 (0.55) |
| Product prescribing error | 4 | 7.87 (2.94 - 21.05) | 7.83 (23.83) | 7.82 (3.44) | 2.97 (1.3) |
| Insurance issue | 4 | 38.95 (14.55 - 104.28) | 38.7 (146.42) | 38.57 (16.92) | 5.27 (3.6) |
| Faeces discoloured | 4 | 12.14 (4.54 - 32.47) | 12.07 (40.59) | 12.06 (5.29) | 3.59 (1.92) |
| Anal incontinence | 3 | 18.09 (5.81 - 56.29) | 18 (48.11) | 17.98 (6.95) | 4.17 (2.5) |
| Faecal volume increased | 3 | 510.62 (160.17 - 1627.84) | 508.13 (1453.34) | 486.4 (184.37) | 8.93 (7.22) |
| Bowel movement irregularity | 3 | 28.48 (9.15 - 88.68) | 28.35 (78.96) | 28.28 (10.93) | 4.82 (3.15) |
| Extra dose administered | 3 | 7.78 (2.5 - 24.19) | 7.74 (17.62) | 7.74 (2.99) | 2.95 (1.28) |
| Defaecation urgency | 3 | 43.3 (13.9 - 134.92) | 43.09 (122.9) | 42.94 (16.59) | 5.42 (3.75) |
| Gastrointestinal sounds abnormal | 3 | 61.09 (19.59 - 190.51) | 60.79 (175.51) | 60.48 (23.35) | 5.92 (4.24) |

PT, preferred term; ROR, reporting odds ratio; PRR, proportional reporting ratio; EBGM, empirical Bayesian geometric mean; EBGM05, the lower limit of the 95% CI of EBGM; IC, information component; IC025, the lower limit of the 95% CI of the IC; CI, confidence interval.

**Supplementary Table S8.** All adverse events meeting the positive signal threshold excluding top 3 combined medication at the PT levels

| PT | Case reports | ROR (95% Cl) | PRR (χ^2^) | EBGM (EBGM05) | IC (IC025) |
| --- | --- | --- | --- | --- | --- |
| Diarrhoea | 229 | 95.53 (57.4 - 159.01) | 94.85 (1384.42) | 94.27 (61.55) | 6.56 (4.89) |
| Abdominal distension | 61 | 9.48 (4.51 - 19.92) | 9.45 (52.91) | 9.45 (5.08) | 3.24 (1.57) |
| Constipation | 59 | 9.3 (1.31 - 66.09) | 9.3 (7.4) | 9.29 (1.8) | 3.22 (1.55) |
| Abdominal pain upper | 54 | 89.7 (12.56 - 640.78) | 89.65 (87.15) | 89.13 (17.2) | 6.48 (4.8) |
| Abdominal pain | 51 | 82.83 (11.6 - 591.49) | 82.79 (80.37) | 82.35 (15.9) | 6.36 (4.68) |
| Muscle spasms | 39 | 8.87 (2.22 - 35.52) | 8.87 (13.95) | 8.86 (2.78) | 3.15 (1.48) |
| Flatulence | 38 | 8.72 (3.62 - 20.97) | 8.7 (34.06) | 8.69 (4.17) | 3.12 (1.45) |
| Product use in unapproved indication | 34 | 8.71 (2.18 - 34.86) | 8.7 (13.63) | 8.7 (2.73) | 3.12 (1.45) |
| Abdominal discomfort | 31 | 8.53 (1.2 - 60.65) | 8.53 (6.65) | 8.53 (1.65) | 3.09 (1.42) |
| Asthenia | 25 | 8.32 (6.42 - 10.78) | 8.11 (369.14) | 8.11 (6.53) | 3.02 (1.35) |
| Therapeutic product effect decreased | 25 | 8.05 (1.13 - 57.2) | 8.05 (6.17) | 8.04 (1.56) | 3.01 (1.34) |
| Product quality issue | 24 | 76.17 (10.67 - 543.7) | 76.14 (73.78) | 75.76 (14.63) | 6.24 (4.56) |
| Malaise | 23 | 75.42 (10.57 - 538.27) | 75.38 (73.03) | 75.01 (14.49) | 6.23 (4.55) |
| Fatigue | 22 | 74.67 (10.46 - 532.94) | 74.64 (72.3) | 74.28 (14.34) | 6.21 (4.54) |
| Headache | 22 | 7.61 (2.45 - 23.61) | 7.6 (17.18) | 7.59 (2.94) | 2.92 (1.26) |
| Therapeutic product effect incomplete | 21 | 7.48 (1.05 - 53.16) | 7.48 (5.61) | 7.48 (1.45) | 2.9 (1.23) |
| Therapy interrupted | 20 | 7.41 (1.04 - 52.67) | 7.41 (5.54) | 7.41 (1.44) | 2.89 (1.22) |
| Patient dissatisfaction with treatment | 19 | 7.32 (1.03 - 52.03) | 7.32 (5.45) | 7.32 (1.42) | 2.87 (1.2) |
| Inability to afford medication | 18 | 7.05 (4.71 - 10.54) | 6.98 (123) | 6.97 (4.98) | 2.8 (1.13) |
| Bowel movement irregularity | 16 | 64.29 (36.41 - 113.53) | 63.93 (740.29) | 63.67 (39.56) | 5.99 (4.32) |
| Faeces discoloured | 16 | 62.64 (8.78 - 446.7) | 62.61 (60.38) | 62.36 (12.05) | 5.96 (4.29) |
| Frequent bowel movements | 15 | 6.94 (2.24 - 21.54) | 6.93 (15.23) | 6.93 (2.69) | 2.79 (1.13) |
| Gastrointestinal sounds abnormal | 15 | 6.86 (5 - 9.42) | 6.75 (191.43) | 6.75 (5.18) | 2.75 (1.09) |
| Dehydration | 14 | 6.79 (1.7 - 27.16) | 6.78 (9.85) | 6.78 (2.12) | 2.76 (1.09) |
| Ill-defined disorder | 13 | 6.67 (2.5 - 17.78) | 6.65 (19.22) | 6.65 (2.93) | 2.73 (1.07) |
| Gastrointestinal motility disorder | 12 | 6.61 (1.65 - 26.46) | 6.6 (9.51) | 6.6 (2.07) | 2.72 (1.06) |
| Product prescribing error | 11 | 6.33 (0.89 - 44.95) | 6.32 (4.48) | 6.32 (1.23) | 2.66 (0.99) |
| Insurance issue | 10 | 6.16 (0.87 - 43.78) | 6.16 (4.32) | 6.16 (1.19) | 2.62 (0.95) |
| Dry mouth | 9 | 55.17 (33.71 - 90.31) | 54.76 (841.53) | 54.57 (36.13) | 5.77 (4.1) |
| Defaecation urgency | 9 | 54.16 (13.5 - 217.25) | 54.11 (103.89) | 53.92 (16.87) | 5.75 (4.08) |
| Gastrointestinal pain | 9 | 51.21 (7.19 - 364.94) | 51.19 (49.04) | 51.02 (9.87) | 5.67 (4) |
| Therapy cessation | 9 | 50.36 (7.07 - 358.86) | 50.34 (48.2) | 50.17 (9.7) | 5.65 (3.97) |
| Anal incontinence | 8 | 50.19 (7.04 - 357.67) | 50.17 (48.03) | 50.01 (9.67) | 5.64 (3.97) |
| Rectal tenesmus | 7 | 5.17 (0.73 - 36.74) | 5.17 (3.36) | 5.17 (1) | 2.37 (0.7) |
| Abdominal pain lower | 7 | 5.03 (0.71 - 35.71) | 5.02 (3.22) | 5.02 (0.97) | 2.33 (0.66) |
| Abdominal tenderness | 6 | 4.98 (0.7 - 35.41) | 4.98 (3.18) | 4.98 (0.97) | 2.32 (0.65) |
| Dyschezia | 6 | 4.64 (0.65 - 32.93) | 4.63 (2.85) | 4.63 (0.9) | 2.21 (0.54) |
| Respiratory disorder | 5 | 35.92 (5.05 - 255.72) | 35.9 (33.85) | 35.82 (6.93) | 5.16 (3.49) |
| Thirst | 5 | 33.02 (4.64 - 235.07) | 33.01 (30.97) | 32.94 (6.38) | 5.04 (3.37) |
| Product availability issue | 5 | 30.02 (4.22 - 213.61) | 30 (27.98) | 29.95 (5.8) | 4.9 (3.23) |
| Presyncope | 4 | 3.93 (0.98 - 15.71) | 3.92 (4.36) | 3.92 (1.23) | 1.97 (0.3) |
| Faeces hard | 4 | 3.64 (0.51 - 25.87) | 3.64 (1.91) | 3.64 (0.71) | 1.86 (0.2) |
| Haemorrhoidal haemorrhage | 4 | 3.64 (0.51 - 25.87) | 3.64 (1.91) | 3.64 (0.71) | 1.86 (0.2) |
| Haemorrhoids | 4 | 3.64 (0.51 - 25.85) | 3.64 (1.91) | 3.64 (0.71) | 1.86 (0.2) |
| Product physical issue | 4 | 3.63 (0.51 - 25.77) | 3.63 (1.9) | 3.62 (0.7) | 1.86 (0.19) |
| Faecaloma | 4 | 3.61 (0.51 - 25.64) | 3.61 (1.88) | 3.61 (0.7) | 1.85 (0.18) |
| Blood sodium decreased | 4 | 3.47 (0.49 - 24.66) | 3.47 (1.76) | 3.47 (0.67) | 1.79 (0.13) |
| Product supply issue | 4 | 3.27 (0.46 - 23.22) | 3.27 (1.57) | 3.27 (0.63) | 1.71 (0.04) |
| Product solubility abnormal | 4 | 3.06 (1.59 - 5.88) | 3.05 (12.4) | 3.05 (1.76) | 1.61 (-0.06) |
| Anorectal discomfort | 3 | 294.04 (186.36 - 463.91) | 291.37 (5394.6) | 285.9 (195.21) | 8.16 (6.49) |
| Oral discomfort | 3 | 24.36 (9.12 - 65) | 24.31 (89.27) | 24.27 (10.68) | 4.6 (2.93) |
| Abnormal faeces | 3 | 23.89 (12.41 - 46) | 23.79 (196.21) | 23.75 (13.73) | 4.57 (2.9) |
| Functional gastrointestinal disorder | 3 | 23.66 (8.87 - 63.15) | 23.62 (86.52) | 23.58 (10.37) | 4.56 (2.89) |
| Sinus headache | 3 | 229.68 (31.86 - 1655.62) | 229.57 (224.18) | 226.16 (43.31) | 7.82 (6.12) |
| Sinus congestion | 3 | 22.86 (3.21 - 162.63) | 22.85 (20.87) | 22.82 (4.42) | 4.51 (2.84) |

PT, preferred term; ROR, reporting odds ratio; PRR, proportional reporting ratio; EBGM, empirical Bayesian geometric mean; EBGM05, the lower limit of the 95% CI of EBGM; IC, information component; IC025, the lower limit of the 95% CI of the IC; CI, confidence interval.

**Supplementary Table S9.** All adverse events meeting the positive signal threshold excluding top 5 combined medication at the PT levels

| PT | Case reports | ROR (95% Cl) | PRR (χ^2^) | EBGM (EBGM05) | IC (IC025) |
| --- | --- | --- | --- | --- | --- |
| Diarrhoea | 208 | 10.92 (9.45 - 12.6) | 9.85 (1670.9) | 9.84 (8.73) | 3.3 (1.63) |
| Constipation | 57 | 8.71 (6.69 - 11.34) | 8.48 (377.46) | 8.48 (6.8) | 3.08 (1.42) |
| Abdominal distension | 56 | 19.29 (14.79 - 25.17) | 18.76 (941.92) | 18.74 (15) | 4.23 (2.56) |
| Abdominal pain upper | 51 | 8.54 (6.47 - 11.29) | 8.35 (330.62) | 8.34 (6.61) | 3.06 (1.39) |
| Abdominal pain | 46 | 7.05 (5.26 - 9.44) | 6.9 (232.96) | 6.9 (5.4) | 2.79 (1.12) |
| Flatulence | 37 | 23.55 (17 - 32.61) | 23.12 (782.43) | 23.08 (17.58) | 4.53 (2.86) |
| Muscle spasms | 34 | 6.46 (4.6 - 9.07) | 6.37 (154.2) | 6.37 (4.79) | 2.67 (1) |
| Product use in unapproved indication | 31 | 2.74 (1.92 - 3.91) | 2.72 (33.8) | 2.72 (2.02) | 1.44 (-0.23) |
| Abdominal discomfort | 30 | 5.29 (3.69 - 7.59) | 5.23 (102.81) | 5.23 (3.86) | 2.39 (0.72) |
| Product quality issue | 24 | 7.63 (5.1 - 11.42) | 7.55 (136.53) | 7.55 (5.39) | 2.92 (1.25) |
| Therapeutic product effect decreased | 24 | 11.11 (7.43 - 16.62) | 10.99 (217.93) | 10.98 (7.84) | 3.46 (1.79) |
| Therapy interrupted | 20 | 7.34 (4.73 - 11.41) | 7.28 (108.41) | 7.27 (5.03) | 2.86 (1.2) |
| Patient dissatisfaction with treatment | 19 | 318.46 (201.81 - 502.54) | 315.34 (5841.43) | 309.41 (211.24) | 8.27 (6.6) |
| Therapeutic product effect incomplete | 18 | 5.15 (3.24 - 8.2) | 5.12 (59.69) | 5.11 (3.47) | 2.35 (0.69) |
| Inability to afford medication | 18 | 44.99 (28.27 - 71.61) | 44.58 (764.92) | 44.46 (30.14) | 5.47 (3.81) |
| Bowel movement irregularity | 16 | 59.75 (36.5 - 97.82) | 59.26 (913.33) | 59.05 (39.1) | 5.88 (4.22) |
| Frequent bowel movements | 15 | 16.53 (9.94 - 27.47) | 16.41 (216.88) | 16.39 (10.71) | 4.03 (2.37) |
| Gastrointestinal sounds abnormal | 14 | 96.47 (56.94 - 163.44) | 95.77 (1305.45) | 95.22 (61.26) | 6.57 (4.91) |
| Faeces discoloured | 14 | 23.98 (14.17 - 40.58) | 23.81 (305.6) | 23.78 (15.31) | 4.57 (2.9) |
| Gastrointestinal motility disorder | 12 | 69.62 (39.42 - 122.95) | 69.19 (803.13) | 68.9 (42.81) | 6.11 (4.44) |
| Ill-defined disorder | 12 | 4.81 (2.72 - 8.48) | 4.78 (35.94) | 4.78 (2.97) | 2.26 (0.59) |
| Product prescribing error | 11 | 9.64 (5.33 - 17.44) | 9.59 (84.64) | 9.58 (5.84) | 3.26 (1.59) |
| Defaecation urgency | 9 | 40.12 (20.83 - 77.29) | 39.94 (340.9) | 39.85 (23.02) | 5.32 (3.65) |
| Dry mouth | 9 | 4.05 (2.11 - 7.81) | 4.04 (20.61) | 4.04 (2.34) | 2.01 (0.35) |
| Insurance issue | 9 | 16.92 (8.79 - 32.57) | 16.84 (134.02) | 16.83 (9.73) | 4.07 (2.41) |
| Gastrointestinal pain | 8 | 22.97 (11.47 - 46.03) | 22.88 (167.2) | 22.85 (12.78) | 4.51 (2.85) |
| Anal incontinence | 8 | 21.07 (10.51 - 42.2) | 20.98 (152.08) | 20.96 (11.72) | 4.39 (2.72) |
| Heart rate increased | 7 | 2.46 (1.17 - 5.16) | 2.45 (6.02) | 2.45 (1.32) | 1.29 (-0.37) |
| Palpitations | 7 | 2.11 (1.01 - 4.43) | 2.11 (4.08) | 2.11 (1.13) | 1.08 (-0.59) |
| Dyschezia | 6 | 45.14 (20.23 - 100.72) | 45.01 (257.49) | 44.89 (22.94) | 5.49 (3.82) |
| Symptom recurrence | 6 | 23.64 (10.6 - 52.71) | 23.57 (129.48) | 23.53 (12.03) | 4.56 (2.89) |
| Rectal tenesmus | 6 | 167.27 (74.75 - 374.31) | 166.76 (978.65) | 165.09 (84.14) | 7.37 (5.7) |
| Abdominal pain lower | 5 | 7.32 (3.04 - 17.62) | 7.31 (27.22) | 7.3 (3.5) | 2.87 (1.2) |
| Respiratory disorder | 5 | 5.76 (2.39 - 13.86) | 5.75 (19.61) | 5.75 (2.76) | 2.52 (0.86) |
| Product availability issue | 5 | 5.16 (2.14 - 12.41) | 5.15 (16.71) | 5.15 (2.47) | 2.36 (0.7) |
| Blood sodium decreased | 4 | 7.96 (2.99 - 21.25) | 7.95 (24.3) | 7.95 (3.5) | 2.99 (1.32) |
| Thirst | 4 | 7.54 (2.83 - 20.13) | 7.53 (22.65) | 7.53 (3.31) | 2.91 (1.24) |
| Presyncope | 4 | 5.66 (2.12 - 15.1) | 5.65 (15.31) | 5.65 (2.49) | 2.5 (0.83) |
| Product physical issue | 4 | 5.52 (2.07 - 14.72) | 5.51 (14.76) | 5.51 (2.42) | 2.46 (0.79) |
| Abdominal tenderness | 4 | 31.46 (11.78 - 83.99) | 31.4 (117.5) | 31.34 (13.78) | 4.97 (3.3) |
| Faeces hard | 4 | 31.42 (11.77 - 83.87) | 31.35 (117.32) | 31.3 (13.76) | 4.97 (3.3) |
| Haemorrhoidal haemorrhage | 4 | 26.36 (9.88 - 70.37) | 26.31 (97.24) | 26.27 (11.55) | 4.72 (3.05) |
| Faecaloma | 4 | 25.61 (9.6 - 68.37) | 25.56 (94.26) | 25.52 (11.22) | 4.67 (3.01) |
| Product solubility abnormal | 4 | 20.91 (7.83 - 55.81) | 20.87 (75.58) | 20.84 (9.17) | 4.38 (2.71) |
| Product supply issue | 4 | 12.29 (4.6 - 32.78) | 12.26 (41.35) | 12.25 (5.39) | 3.62 (1.95) |
| Sinus congestion | 3 | 8.23 (2.65 - 25.56) | 8.22 (19.03) | 8.22 (3.19) | 3.04 (1.37) |
| Oral discomfort | 3 | 7.51 (2.42 - 23.32) | 7.5 (16.9) | 7.5 (2.91) | 2.91 (1.24) |
| Haemorrhoids | 3 | 5.41 (1.74 - 16.79) | 5.4 (10.76) | 5.4 (2.09) | 2.43 (0.77) |
| Anorectal discomfort | 3 | 29.77 (9.58 - 92.48) | 29.72 (83.13) | 29.67 (11.49) | 4.89 (3.22) |
| Functional gastrointestinal disorder | 3 | 25.1 (8.08 - 77.95) | 25.06 (69.2) | 25.02 (9.69) | 4.65 (2.98) |
| Faecal volume increased | 3 | 179.82 (57.59 - 561.49) | 179.54 (526.87) | 177.6 (68.5) | 7.47 (5.8) |
| Proctalgia | 3 | 16.59 (5.34 - 51.51) | 16.56 (43.83) | 16.55 (6.41) | 4.05 (2.38) |
| Abnormal faeces | 3 | 11.2 (3.61 - 34.77) | 11.18 (27.8) | 11.18 (4.33) | 3.48 (1.81) |

PT, preferred term; ROR, reporting odds ratio; PRR, proportional reporting ratio; EBGM, empirical Bayesian geometric mean; EBGM05, the lower limit of the 95% CI of EBGM; IC, information component; IC025, the lower limit of the 95% CI of the IC; CI, confidence interval.
